# Comparing fentanyl and dexmedetomidine as adjuvants to bupivacaine for spinal anesthesia in appendectomy: effects on peritoneal symptoms – a randomized clinical trial

**DOI:** 10.1186/s13741-025-00618-5

**Published:** 2025-11-14

**Authors:** Ayman Mohamady Eldemrdash, Tarek S. Hemaida, Mohammed Ahmed Alazhary, Ahmed Abdelrahim Mahmoud, Ahmed Mohsen Hagag, Soudi S. Hammad

**Affiliations:** Anesthesiology, Surgical Intensive Care and Pain Medicine, Faculty of Medicine, Aswan University, Aswan, 82511 Egypt

**Keywords:** Fentanyl, Dexmedetomidine, Bupivacaine, Appendectomy, Peritoneal Symptomatic Effects

## Abstract

**Background:**

Peritoneal symptoms, including visceral pain, abdominal discomfort, and vagal responses (e.g., nausea, bradycardia), are common during abdominal surgeries under spinal anesthesia. This study compared intrathecal dexmedetomidine and fentanyl for their effectiveness in controlling these symptoms during appendectomy.

**Methods:**

This randomized, double-blinded clinical trial included 150 patients of the American Society of Anesthesiologists I, II physical status scheduled for emergency open appendectomy. Participants were randomly assigned to receive either intrathecal dexmedetomidine (5 μg, Group D) or fentanyl (25 μg, Group F), both combined with 0.5% hyperbaric bupivacaine.

**Results:**

Dexmedetomidine significantly reduced the incidence of peritoneal symptoms compared to fentanyl: abdominal discomfort (9.5% vs. 33.3%),

visceral pain (10.8% vs. 53.3%), nausea (9.5% vs. 34.7%), and vomiting (6.8% vs.34.7%) (*P* < 0.001). The time to first rescue analgesia was significantly longer in the dexmedetomidine group (396 vs. 243 min; *P* < 0.001). Bradycardia was more frequent in group D (25.7% vs. 1.3%, *P* < .001); no cases of respiratory depression were observed. Hypotension occurred slightly more frequently in group D, whereas shivering was more prevalent in group F; however, neither difference reached statistical significance. The VAS was significantly higher in group F than in group D at four and six hours postoperatively (*P* < 0.001).

**Conclusions:**

Dexmedetomidine provides superior peritoneal symptom control and prolonged analgesia compared to fentanyl as an intrathecal adjuvant in spinal anesthesia for appendectomy. Despite a higher incidence of bradycardia, its opioid-sparing benefits and overall safety make it a valuable alternative, particularly for procedures involving significant visceral manipulation.

## Introduction

Acute appendicitis is one of the most common indicators for emergency abdominal surgery worldwide, contributing significantly to emergency department admissions (Cervellin et al. 2016). Although general anesthesia remains the standard approach due to its advantages in muscle relaxation and reduced aspiration risk, spinal anesthesia is gaining popularity for its benefits, including faster recovery, shorter hospital stays, earlier return to oral intake, and lower healthcare costs. (Medina Donoso et al. 2021). However, spinal anesthesia presents unique challenges in abdominal surgeries, particularly in controlling peritoneal symptoms such as visceral pain, abdominal discomfort, nausea, vomiting, bradycardia, and hypotension (Raddad et al. 2024).

To enhance the efficacy of spinal anesthesia, intrathecal adjuvants are commonly added to improve sensory blockade, prolong postoperative analgesia, and enhance patient comfort (Gupta et al. 2011). Among these, intrathecal opioids, particularly fentanyl, have demonstrated significant efficacy in reducing visceral and somatic pain (El-Fakharany and El-Den Shamlol 2023). However, fentanyl is associated with side effects, such as pruritus, respiratory depression, and postoperative nausea and vomiting (PONV), limiting its broad applicability (Khan et al. 2015).

Dexmedetomidine, a highly selective alpha 2-adrenergic agonist, has emerged as a promising alternative to opioids due to its ability to induce deep sensory and motor block, provide sedation, and reduce anxiety, while minimizing respiratory depression (Minagar et al. 2019). Additionally, dexmedetomidine synergistically enhances bupivacaine’s effects, leading to a denser and longer-lasting spinal block (Kanazi et al. 2006).

Although dexmedetomidine has been widely studied as an intrathecal adjuvant in elective procedures, such as cesarean sections and orthopedic surgeries, its role in emergency abdominal surgeries, particularly appendectomy, remains underexplored (Divya et al. 2021). Furthermore, the management of peritoneal symptoms under spinal anesthesia remains a relatively unexamined area, particularly in an emergency surgical setting. This study addresses this gap by focusing on peritoneal symptoms, an often-overlooked clinical challenge in spinal anesthesia.

We hypothesized that intrathecal dexmedetomidine would provide superior peritoneal symptom control, longer postoperative analgesia, and fewer opioid-related side effects compared to fentanyl in patients undergoing emergency open appendectomy.

Patients and Methods.

### Study design and ethical approval

This randomized, double-blinded clinical trial was conducted at Aswan University Hospitals, Egypt, were included in the study between May 2024 to January 2025. The study was approved by the Institutional Review Board (IRB)of Aswan University Hospitals (Approval Number: Asw.U./823/7/23) and was registered on ClinicalTrials.gov (NCT06386783, Principal investigator: Soudy Salah Hammad, Registration Date: February 5, 2024).

Written informed consent was obtained from all participants before enrollment. If the participant was unable to provide consent, written informed consent was obtained from their legal representative.

### Participants and eligibility criteria

A total of 150 adult patients (aged 18–60 years) with American Society of Anesthesiologists (ASA) I or II physical status scheduled for emergency open appendectomy in Aswan University Hospitals, Egypt. Only early or uncomplicated appendicitis included; perforated/gangrenous excluded. Also, our team excluded cases developing infection at the injection site, coagulopathy or anticoagulation, congenital anomalies in the lower spine, active CNS disease, as well as prior allergic reactions to local anesthetics.

### Randomization and blinding

The patients were randomly assigned to two groups (n = 75 per group) using a computer-generated randomization sequence. An independent anesthesiologist, not involved in patient monitoring or data collection, performed the randomization. To ensure blinding, participant codes were secured in opaque, sealed envelopes. Both the participants as well as the outcome assessor were unaware of the medications being administered. All intrathecal drugs were prepared in identical syringes to maintain blinding.

#### Group allocation

- **Group D (Dexmedetomidine,*n*** =** 75)**: administered 5 μg of Dexmedetomidine with 4 ml of 0.5% hyperbaric bupivacaine hydrochloride.
- **Group F (Fentanyl,** ***n*** =** 75)**: got 25 μg of Fentanyl together with 4 ml of 0.5% hyperbaric bupivacaine HCl.

### Drug preparation

To ensure precise dosing, uniform volume, and blinding, all intrathecal solutions were prepared in identical 0.5 mL syringes under sterile conditions by an independent anaesthesiologist not involved in patient management or data collection.

### Fentanyl group

A 0.5 mL solution containing 25 µg fentanyl was prepared as follows:

Fentanyl concentration: 100 µg in 2 mL (50 µg/mL).

Required dose: 25 µg.

Preparation: 0.5 mL of fentanyl solution (containing 25 µg) was drawn into the syringe.

### Dexmedetomidine group

A 0.5 mL solution containing 5 µg dexmedetomidine was prepared as follows:

Dexmedetomidine concentration: 200 µg in 2 mL (100 µg/mL).

Required dose: 5 µg.

Preparation:

- Withdraw 1 mL (100 µL) of dexmedetomidine from the ampule and dilute it with 9 mL of normal saline in a sterile syringe.
- This creates a diluted solution with a final concentration of 10 µg/ml.
- From this diluted solution, withdraw 0.5 ml, which now contains the required 5 µg dexmedetomidine.

The doses of fentanyl (25 µg) and dexmedetomidine (5 µg) were selected based on previous studies demonstrating their efficacy in enhancing spinal anesthesia while maintaining an acceptable safety profile. The fentanyl dose provides prolonged sensory blockade and intraoperative analgesia (El-Fakharany and El-Den Shamlol 2023; Khan et al. 2015; Divya et al. 2021). Dexmedetomidine, although not yet FDA-approved for intrathecal use, has been widely investigated in clinical trials. The 5 µg dose was chosen based on literature indicating that lower doses (≤ 5 µg) provide significant prolongation of anesthesia and better hemodynamic stability compared to higher doses, which are more likely to cause bradycardia and hypotension (Divya et al. 2021).

All patients underwent a standard open appendectomy via a McBurney right iliac fossa incision. All intrathecal injections were administered in sitting position and by 25G Quincke spinal needle after confirming free cerebrospinal fluid (CSF), at site: L3–L4 or L4–L5 and injection was 4 mL of 0.5% hyperbaric bupivacaine with either fentanyl 25 µg or dexmedetomidine 5 µg, injected over 10–15 s, no barbotage and patients immediately placed supine (Jun et al. 2014).

### Intraoperative and postoperative monitoring

Patients were continuously monitored throughout surgery for heart rate (HR), noninvasive blood pressure (NIBP), electrocardiogram (ECG), and peripheral oxygen saturation (SpO2).

Hypotension (> 20% decrease from baseline) was treated with 200 mL Ringer’s lactate and IV ephedrine (6 mg). Bradycardia (HR < 50 bpm) was managed with IV atropine (0.5 mg). Respiratory depression (RR < 9 breaths/min, SpO₂ < 90%) was monitored. Oxygen supplementation (via facemask), jaw thrust, and bag-mask ventilation if required, and naloxone 40 μg IV boluses for opioid-induced depression.

Sensory block was assessed using the pinprick test, while motor block was evaluated with the Modified Bromage Score;0 (full movement) to 3 (complete motor block), and rescue analgesia criteria by Visual Analog Scale (VAS); 0 (no pain) to10 (worst imaginable pain), respectively if > 4, patient was treated by intramuscular diclofenac (75 mg). Patients were monitored for six hours postoperatively in the post-anesthetic care unit (PACU).

### Primary outcome

Incidence of peritoneal symptoms (visceral pain, abdominal discomfort, nausea, vomiting, bradycardia, hypotension).

### Secondary outcomes

Time to first rescue analgesia, VAS scores at 1, 2, 4, 6 h, adverse effects (shivering, pruritus), and motor block duration.

### Sample size calculation

The sample size was calculated using G*Power 3.1.9.4 software. Based on a previous study reporting 30% incidence of nausea in one group vs. 10% in the other (Agrawal et al. 2016, Efficacy of intrathecal lipophilic versus lipophobic opioids) (Agrawal et al. 2016). The following parameters were used: Alpha (type I error): 0.05; Power (1–beta, type II error): 0.80. Effect size: Derived from the expected difference in nausea rates. The required sample size was 62 patients per group. To account for potential dropouts, 75 patients were included in each group, yielding a total of 150 participants.

### Statistical analysis

We conducted statistical analysis utilizing SPSS version 26 (IBM Inc., Chicago, IL, USA). Data normality was assessed using the Shapiro–Wilk test. Quantitative variables are presented as mean ± SD for normally distributed data or as median [IQR] for non-normal data. Parametric data were compared using an independent Student’s *t* test; non-parametric data using–Mann–Whitney *U* test. Categorical variables were expressed as n (%) and compared using Chi-square or Fisher’s exact test. *p* < 0.05 was considered statistically significant.

## Results

**Fig. 1:**
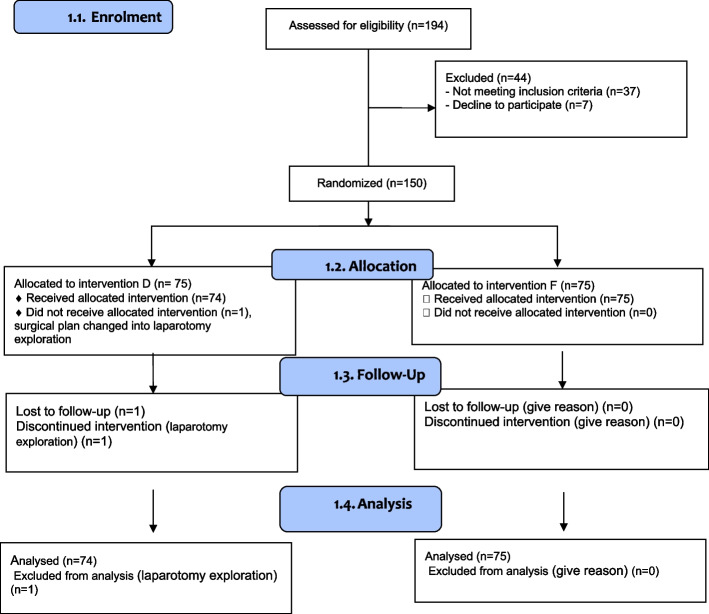
CONSORT Flowchart D: Dexmedetomidine, F: Fentanyl

A total of 194 patients were screened, of whom 44 were excluded due to not meeting inclusion criteria (*n* = 37) or declining participation (*n* = 7). The remaining 150 patients were randomized into two groups: Group D (Dexmedetomidine, *n* = 75) and Group F (Fentanyl, n = 75). One patient in Group D was excluded post-randomization due to a surgical plan change, leaving 74 in Group D and 75 in Group F for analysis (Fig. 1).

There were no significant differences between the two groups in age, operative time, ASA classification, or smoking status (Table 1).

**Table 1:** Demographic data

		**Group D (*n* = 74)**	**Group F (*n* = 75)**	**P**
Age (years, mean ± SD)		27.3 ± 9.4	28.4 ± 9.8	0.48
Gender (M/F)		42/32	44/31	0.88
Weight (kg, mean ± SD)		71.8 ± 9.5	72.5 ± 10.1	0.67
Height (cm, mean ± SD)		169.5 ± 7.6	170.2 ± 7.4	0.52
Apfel PONV score (median [IQR])		2 [1–3]	2 [1–3]	0.72
Operative time (min)		52.7 ± 3	52.6 ± 2.9	0.91
Fluids infused (mL, mean ± SD)		1095 ± 220	1120 ± 235	0.44
Ephedrine dose (mg, mean ± SD)		8.4 ± 2.1	7.9 ± 2.3	0.32
Smoking status (n, %)		8 (10.8%)	13 (17.3%)	0.36
ASA	I	59 (79.7%)	56 (74.7%)	0.58
	II	15 (20.3%)	19 (25.3%)	

Data are presented as mean ± SD, median [IQR], or frequency (%). D: Dexmedetomidine, F: Fentanyl, ASA: American Society of Anesthesiologists, PONV: postoperative nausea and vomiting

The incidence of peritoneal symptoms was significantly lower in group D compared to Group F: abdominal discomfort (9.5% vs. 33.3%), visceral pain (10.8% vs. 53.3%), nausea (9.5% vs. 34.7%), and vomiting (6.8% vs. 34.7%). The total peritoneal symptom score was significantly lower in Group D compared to Group F (mean difference: −0.8,95%CI: [−1.2 to −0.3], *P* < 0.001).

Bradycardia was significantly more frequent in Group D (25.7% vs. 1.3%, *P* < 0.001), while pruritus occurred more often in Group F (21.3% vs. 2.7%, *P* < 0.001). Hypotension was slightly higher in Group D, and shivering was more common in Group F; however, neither difference reached statistical significance (Table 2). No cases of respiratory depression were observed in either group.

**Table 2:** Adverse events/peritoneal symptom incidence: -

	**Group D (*n* = 74)**	**Group F (*n* = 75)**	**Effect size** **[CI 95%]**	**P**
Abdominal discomfort (n, %)	7 (9.5%)	25 (33.3%)	0.2 [0.08- 0.52]	0.0001 *
Visceral pain (n, %)	8 (10.8%)	40 (53.3%)	0.1 [0.04- 0.25]	0.0001 *
Nausea (n, %)	7 (9.5%)	26 (34.7%)	0.19 [0.79- 0.49]	0.0001 *
Vomiting (n, %)	5 (6.8%)	26 (34.7%)	0.13 [0.05- 0.38]	0.0001 *
Bradycardia (n, %)	19 (25.7%)	1 (1.3%)	25.6 [3.3- 196]	0.0001 *
Hypotension (n, %)	26 (35.1%)	15 (20.0%)	2.17 [1.03- 4.5]	0.059
Respiratory depression	0	0	NA	0.999
Shivering (n, %)	16 (21.6%)	25 (33.3%)	0.5 [0.26- 1.15]	0.15
Pruritus (n, %)	2 (2.7%)	16 (21.3%)	0.1 [0.02–0.46]	0.001 *
Total peritoneal symptoms (mean ± SD)	0.97 ± 1.2	1.78 ± 1.7	- 0.55 [- 0.8- −0.22]	0.0007*
Mean difference [CI 95%]	−0.8 [−1.2- −0.3]			

Data are presented as mean ± SD or frequency (%). * Significant p value < 0.05, D: Dexmedetomidine, F: Fentanyl, CI: confidence interval

**Fig. 2:**
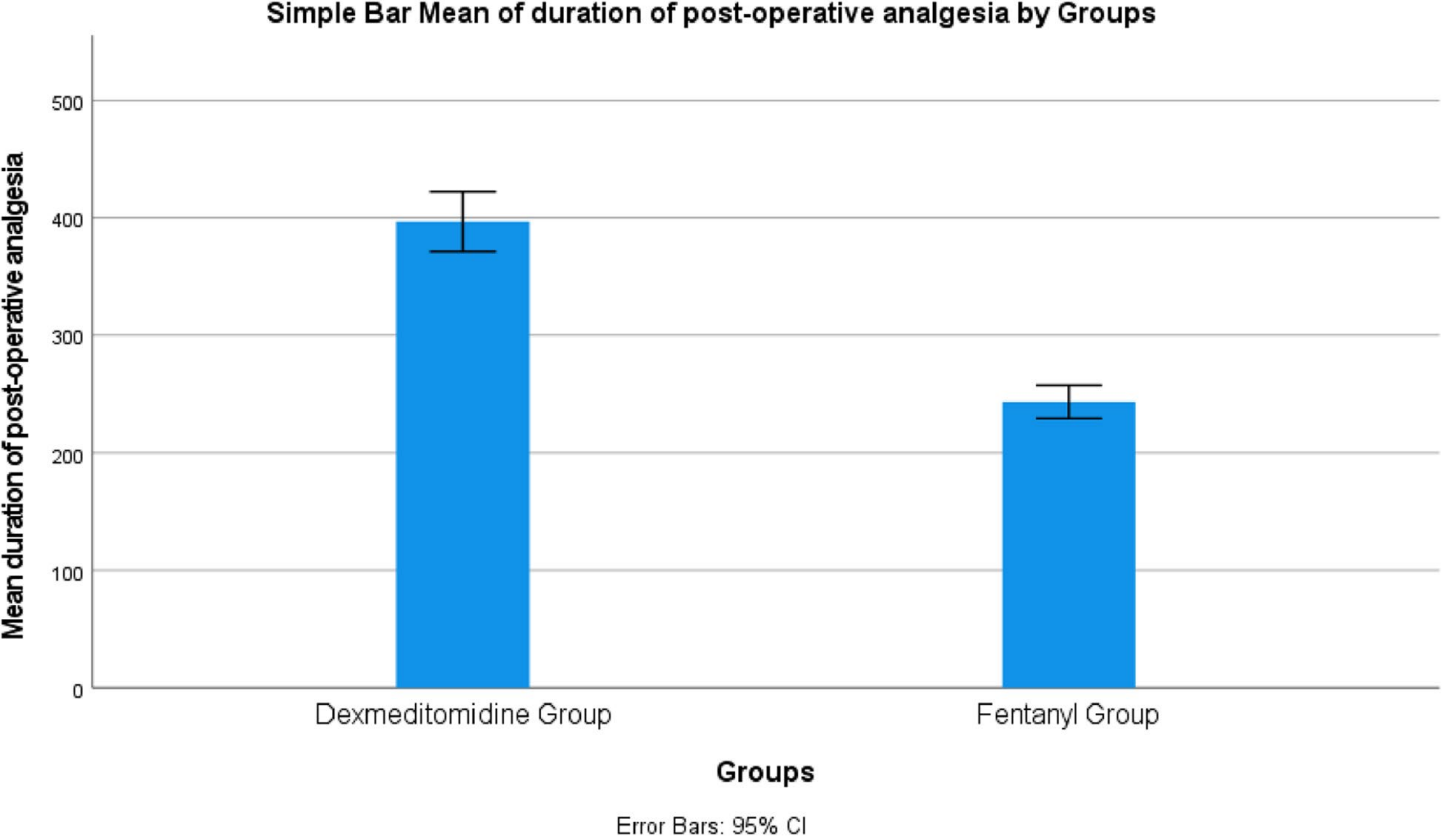
Duration of postoperative analgesia

Patients in Group D experienced significantly longer postoperative analgesia compared to Group F: time to first rescue analgesia was (396.9 ± 109 vs. 243.2 ± 61.3 min; *P* < 0.001) and time to full sensory recovery was (298.9 ± 35.3 vs. 209.1 ± 26.3 min; *P* < 0.001) (Table 3, Fig. 2).

**Table 3:** Time-to-event measures: -

	**Group D (n = 74)**	**Group F (n = 75)**	**P**
Time to rescue first analgesic dose (min)	396. 9 (109)	243.2 (61.3)	0.0001*
Mean difference [CI 95%]	153.69 [124.8- 182.5]		
Effect size [CI 95%]	1.74 [1.3- 2.1]		
time to regain full sensory function (min)	298.9 (35.3)	209.1 (26.3)	0.001*
Mean difference [CI 95%]	89.7 [79.7- 99.8]		
Effect size [CI 95%]	2.88 [2.4- 3.3]		

Data are presented as mean ± SD or frequency (%). * Significant p value < 0.05, D: Dexmedetomidine, F: Fentanyl, CI: confidence interval

**Fig. 3:**
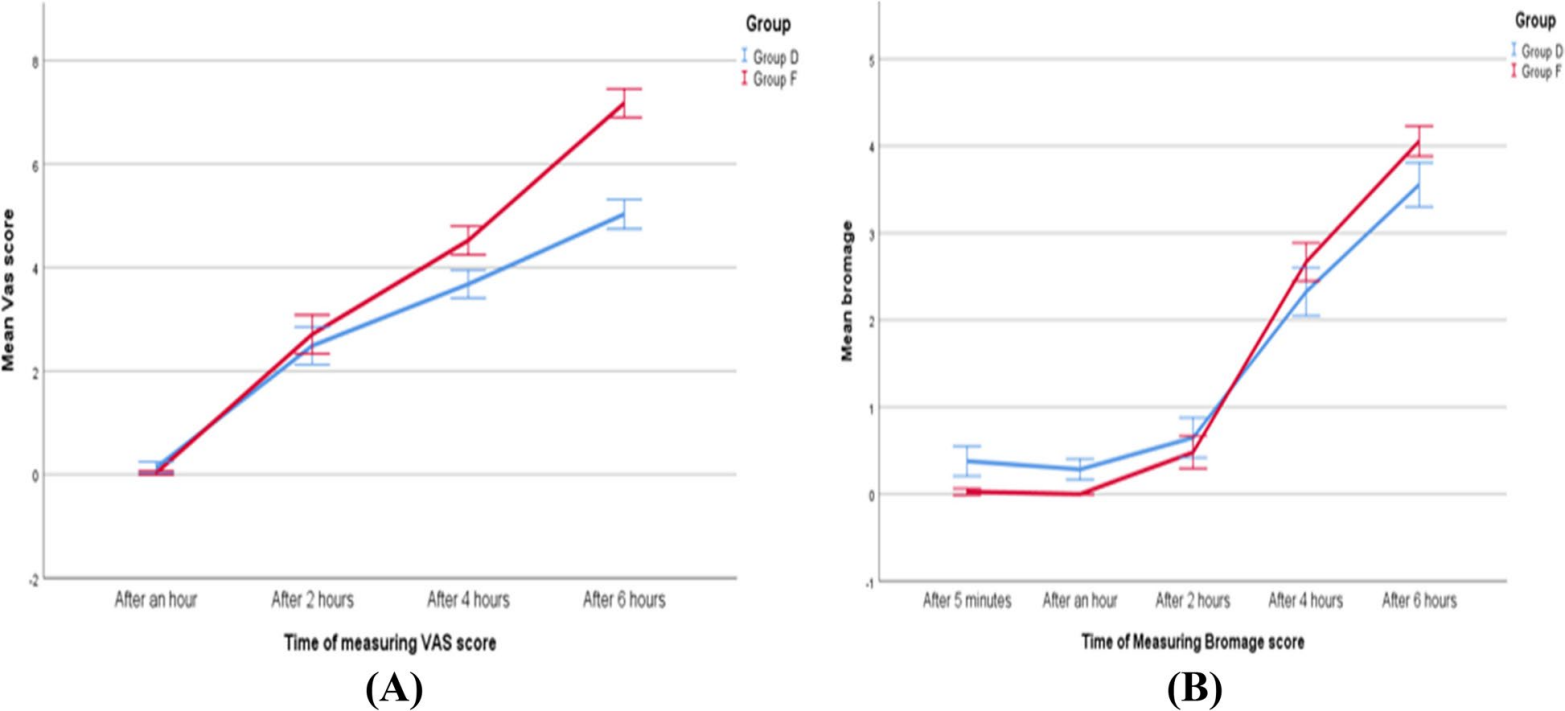
Line graphs of VAS and Bromage score

VAS scores were comparable between the groups at 1 and 2 h postoperatively, but significantly higher in Group F at 4 and 6 h (*P* < 0.001). Bromage scores (motor blockade) were higher in Group D at 5 min and 1 h (*P* < 0.001), with no differences at 2 and 4 h. But at 6 h, Group F showed faster motor recovery, indicating a shorter motor blockade duration with Fentanyl (Fig. 3).

## Discussion

This randomized clinical trial compared intrathecal dexmedetomidine and fentanyl as adjuvants to bupivacaine in patients undergoing open appendectomy under spinal anesthesia, focusing on peritoneal symptoms, postoperative analgesia, and recovery outcomes. Our findings demonstrate that dexmedetomidine provided superior control of peritoneal symptoms, prolonged postoperative analgesia, and reduced opioid-related adverse effects, although it was associated with a higher incidence of bradycardia.

Peritoneal symptoms such as visceral pain, nausea, vomiting, and abdominal discomfort represent a significant clinical challenge during spinal anesthesia for abdominal surgery. In our study, dexmedetomidine markedly reduced these symptoms compared with fentanyl. This aligns with previous studies indicating that dexmedetomidine, a selective α2-adrenergic agonist, enhances sensory blockade by inhibiting C-fibres transmission and modulating dorsal horn nociception, thereby improving visceral pain suppression (Khan et al. 2015; Gautam et al. 2018; Khosravi et al. 2020). In contrast, intrathecal fentanyl primarily targets somatic pain via opioid receptors and is more frequently associated with pruritus and postoperative nausea (Liu and Zhao 2020).

Our results also revealed a significantly longer time to first rescue analgesia in the dexmedetomidine group (396 vs. 243 min), consistent with prior evidence that low-dose intrathecal dexmedetomidine prolongs both sensory block duration and postoperative analgesia (Divya et al. 2021; Sun et al. 2015). The lower VAS scores at 4 and 6 h postoperatively further support its sustained analgesic effect. These results indicate that dexmedetomidine provided prolonged pain relief compared to fentanyl. While motor blockade was initially denser with dexmedetomidine, recovery was faster in the fentanyl group, which may facilitate earlier mobilization. Thus, the choice of adjuvant may be influenced by the balance between prolonged analgesia and the need for rapid motor recovery (Kanazi and Aouad 2006).

Hemodynamically, bradycardia occurred more frequently with dexmedetomidine but was effectively managed with atropine and did not result in adverse sequelae. Hypotension rates were slightly higher but not statistically significant, consistent with prior evidence suggesting that low-dose dexmedetomidine (≤ 5 µg) minimizes major hemodynamic compromise (Talke and Tayefeh 1997). Importantly, no cases of respiratory depression were reported in either group, reinforcing the safety of both adjuvants at the studied doses. Additionally, shivering rates were comparable, suggesting that both adjuvants provided thermoregulatory stability (Usta and Gozdemir 2011).

From a clinical perspective, dexmedetomidine offers meaningful advantages in appendectomy, where visceral manipulation is prominent. Its ability to reduce peritoneal symptoms and prolong analgesia may improve patient comfort, reduce intraoperative supplemental analgesic requirements, and potentially decrease opioid consumption postoperatively. Nevertheless, clinicians must remain vigilant for bradycardia and manage it promptly.

This study has several limitations.

First, the follow-up period was limited to 6 h, potentially missing late-onset complications or analgesic requirements. Second, peritoneal symptoms were assessed using subjective measures, which may introduce observer bias. Third, ASA III–IV patients were excluded, limiting generalizability to higher-risk populations. The absence of a placebo (bupivacaine-only) group prevents direct assessment of absolute adjuvant benefits. Finally, the sample size calculation was powered on the expected difference in nausea; some secondary outcomes may require larger samples to precisely estimate effect sizes. Future multicentre trials with longer follow-up and broader patient inclusion are warranted.

## Conclusion

In this randomized clinical trial, intrathecal dexmedetomidine was associated with a lower incidence of peritoneal symptoms and a longer time to first rescue analgesia compared with intrathecal fentanyl. However, dexmedetomidine was also associated with a higher incidence of bradycardia; these results are limited to the doses and population studied and should be confirmed in larger, multicentre trials.

## Data Availability

The datasets generated and analyzed during the current study are not publicly available due to institutional restrictions, but are available from the corresponding author upon reasonable request.

## Declarations

**Ethical approval and consent to participate**

This study was approved by the ethics committee of Aswan University Hospital (approval code:/823/7/23). All patients signed a written informed consent before inclusion in this study.
